# Incidence and clinical features of endometriosis in 2484 consecutive female patients undergoing appendectomy for suspected appendicitis—a retrospective analysis

**DOI:** 10.1007/s00423-024-03328-6

**Published:** 2024-04-29

**Authors:** M. Schrempf, M.-A. Kirmair, A. Mair, M. Hoffmann, C. Dannecker, M. Anthuber, L. Anthuber

**Affiliations:** 1 Department of General, Visceral and Transplantation Surgery, University Hospital Augsburg, Stenglinstrasse 2, 86156 Augsburg, Germany; 2 Department of Gynecology and Obstetrics, University Hospital Augsburg, University of Augsburg, Augsburg, Germany

**Keywords:** Appendicitis, Emergency surgery, Endometriosis, Appendectomy

## Abstract

**Introduction:**

Endometriosis is a common condition affecting 5 to 10% of women of childbearing age. The true incidence of endometriosis of the appendix is currently unknown. Since symptoms often overlap with those of acute appendicitis, endometriosis of the appendix presents a diagnostic challenge in the emergency department.

This large retrospective study investigates the incidence and perioperative clinical, radiologic, and laboratory findings, as well as possible differences between patients with and without endometriosis.

**Methods:**

Data from consecutive patients who underwent appendectomy for suspected appendicitis without a history of endometriosis were analyzed. Perioperative clinical, laboratory, perioperative, and histopathologic findings were compared between women with and without endometriosis.

**Results:**

Between January 2008 and June 2023, 2484 consecutive patients without a history of endometriosis underwent urgent appendectomy for suspected appendicitis. Endometriosis was detected on histopathologic examination in 17 (0.7%) patients. Signs of appendicitis were found less frequently on ultrasound in the endometriosis group compared to the non-endometriosis group (23.4% vs. 61.5%; *p* = 0.002; OR = 0.193; 95% CI 0.063–0.593). There were no differences in physical examination findings, duration of symptoms, degree of inflammation, surgical outcomes, or complication rates.

**Conclusion:**

The incidence of endometriosis of the appendix in patients undergoing appendectomy for suspected appendicitis was higher than suggested by data from autopsy series and populations with biopsy-proven endometriosis. Patients with endometriosis of the appendix were less likely to have a positive ultrasound finding, but perioperative and histopathologic findings and severity of inflammation did not differ from patients without endometriosis, presenting diagnostic challenges for clinicians.

## Introduction

Endometriosis is a common disease in woman of reproductive age, with an estimated prevalence of approximately 5 to 10 percent of the overall population [7, 24]. The assessment of prevalence in asymptomatic women or the general population is difficult because definitive diagnosis usually requires laparoscopy with biopsy histopathologic examination [16]. The reported prevalence in symptomatic populations reaches up to 50 percent in woman with infertility and up to 47 percent in woman and adolescent females with pelvic pain [11, 16, 21, 24]. The most common organs and anatomic sites affected by endometriosis are the ovaries, vesico- and recto-uterine pouch, uterosacral and posterior broad ligaments, uterus, fallopian tubes, rectum and sigmoid colon [3, 14, 18, 20]. Endometriosis of the gastrointestinal tract can cause diarrhea, constipation, dyschezia, bowel cramping, and in rare cases rectal bleeding [4, 13]. Endometriosis of the appendix is a rare manifestation but it can present a diagnostic challenge in woman with acute abdominal pain. While endometriosis usually presents with chronic or recurrent symptoms, or both, endometriosis of the appendix in the acute setting is of particular interest because not only can it mimic acute appendicitis, but it has been postulated that endometriosis of the appendix can cause acute appendicitis [25]. Several cases of endometriosis as underlying cause for acute appendicitis including uncomplicated and complicated appendicitis have been described [2, 12, 14, 17, 22, 25].

In a large retrospective review of single institution pathology reports of patients with biopsy proven endometriosis, appendiceal endometriosis is present in than 0.2% of cases (3 of 1376) [20]. In a historic series of 50,000 appendectomy specimens from the 1950s an incidence of 0.054% was reported [10].

Data on the frequency, clinical, laboratory and radiologic features of endometriosis as an underlying cause of appendicitis or endometriosis in patients presenting with suspected appendicitis is rare und probably underreported. Currently it is unknown if symptoms, diagnostic findings, and perioperative findings vary from women without endometriosis.

The purpose of this study was to determine the frequency of endometriosis in women undergoing appendectomy for suspected appendicitis and to compare perioperative findings between patients with postoperative histopathologically proven endometriosis and a group without endometriosis to determine characteristics that might facilitate identification of patients with endometriosis in the emergency setting. In this study, we present data from a large retrospective single-institution study of woman undergoing appendectomy for suspected appendicitis. The incidence of endometriosis and the clinical, radiologic, laboratory, and perioperative and histologic characteristics are presented and compared between the two study groups.

## Methods

This study was conducted at the Department of General Surgery, Visceral and Transplant Surgery at the University Hospital Augsburg, Germany as a single center retrospective study. The study was approved by the Ethics Committee of the Ludwig Maximilians University (LMU), Munich (reference number 23–0500) and conducted in accordance with the Declaration of Helsinki.

### Study population and definitions

We identified all women without a history of endometriosis who underwent emergency surgery for suspected appendicitis at our institution between January 2008 and June 2023 from the institutional electronic database. Electronic health records were reviewed, and perioperative data were extracted. Complications, comorbidities, operative data and patient characteristics were collected from the database, including age, ASA status, BMI, preoperative symptoms, preoperative CRP, leukocyte and bilirubin levels, radiologic findings, intraoperative findings, operating time, percentage of laparoscopic procedures, histopathology results, complication rate and length of hospital stay. All histopathologic reports were reviewed, and patients were divided into two groups. The endometriosis group consisted of patients with endometriosis in the appendectomy specimen and a non-endometriosis group without histopathological findings of endometriosis in the surgical specimen.

### Statistical analysis

Continuous data is presented as mean ± standard deviation or median with interquartile range, depending on distribution. Categorical data is presented as numbers with percentages. Continuous variables were compared using the independent t-test and the Mann–Whitney-U test depending on distribution. Categorical data was compared using the χ2 test. Fisher’s exact test was used for categorical data when the requirements for χ2 test were not met. A two-sided *P* < 0.05 was considered significant. Clinical, laboratory, radiologic and histopathologic findings were tested for association with endometriosis in a univariate analysis. Furthermore, complication rates were compared between the two groups. Statistical analyses were undertaken using SPSS® for macOS®, version 28 (IBM, Armonk, New York, USA).

## Results

We identified 2484 consecutive female patients (age ≥ 16 years) who underwent urgent appendectomy for suspected appendicitis between January 2008 and June 2023. In 17 (0.7%) patients without a prior history of endometriosis, histopathologic examination of the removed appendix revealed endometriosis.

Demographic and preoperative findings were compared between the two study groups. The results are shown in Table 1. There were no differences with regard to age, BMI, clinical findings on physical examination and duration of symptoms between patients with and without endometriosis. Appendicitis was suspected on ultrasound in 23.4% of patients in the endometriosis group and 61.5% in the non-endometriosis group (*p* = 0.002; OR = 0.193; 95% CI 0.063–0.593). Visualization of the appendix and suspected appendicitis on ultrasound was less frequent in the endometriosis group compared to patients without endometriosis (17.6% vs. 51.2%, *p* = 0.006, OR 0.204; 95% CI 0.058–0.712). Preoperative white blood cell count (WBC), C-reactive protein (CRP) and bilirubin levels did not differ between groups.

**Table 1:** Demographic and preoperative characteristics

	Endometriosis (*n* = 17)	No endometriosis (*n* = 2467)	*p*
Age	37.0 ± 16.0	36.8 ± 18.3	0.97
BMI	22.8 ± 5.0	24.8 ± 5.5	0.12
Pain migration			
- Yes	2 (11.8)	508 (20.6)	0.55
- No	15 (88.2)	1959 (79.4)	
Guarding			
- Yes	6 (35.3)	948 (38.4)	0.79
- No	11 (64.7)	1519 (61.6)	
Rigiditys			
- Yes	2 (11.8)	193 (7.8)	0.39
- No	15 (88.2)	2274 (92.2)	
Rebound tendernesss			
- Yes	5 (29.4)	631 (25.6)	0.78
- No	12 (70.6)	1836 (74.4)	
Nausea and / or vomiting			
- Yes	6 (35.3)	667 (27.0)	0.42
- No	11 (64.7)	1800 (73.0)	
Duration of symptoms prior to presentation at ED			
- < 24 h	9 (60.0)	1160 (49.3)^‡^	0.41^‡^
- ≥ 24 h	6 (40.0)	1192 (50.7)^‡^	
- unknown		115	
WBC (per nl)	14.2 ± 6.8	12.7 ± 4.6	0.38
CRP (mg/l)	52 ± 66	50 ± 69	0.90
Bilirubin (mg/dl)	0.55 ± 0.23	0.65 ± 0.51	0.49
Appendicitis suspected on ultrasound			
- Yes	4 (23.4)	1475 (61.5)	0.002
- No	13 (76.5)	924 (38.5)	
Visualization of appendix achieved, and appendicitis suspected on ultrasound			
- Yes	3 (17.6)	1229 (51.2)	0.006
- No	14 (82.4)	1170 (48.8)	
Perforation suspected on ultrasound			
- Yes	0	136 (5.7)	*p* = 0.62
- No	17 (100)	2263 (94.3)	

Data are mean ± SD or *n* (%) or median (IQR)

*IQR* Interquartile range; *SD* Standard deviation; *BMI* Body mass index; CRP C-reactive protein; *WBC* White blood cell count

^‡^ Percentages and p-value refer to subgroup of patients with known duration of symptoms

Surgical approach, operating time, length of postoperative stay, rate of perforation, rate of severe complications (Clavien Dindo grade III or higher) and the Comprehensive Complication Index (CCI®) did not differ between groups (Table 2).

**Table 2:** Intraoperative and postoperative findings

	Endometriosis (*n* = 17)	No endometriosis (*n* = 2467)	*p*
Operating time (min)	56 ± 14.8	63 ± 29.9	0.39
Surgical approach			
- Laparoscopic	17 (100)	2289 (92.8)	0.63
- Open or conversion	0	178 (7.2)	
Length of postoperative stay	2.45 (IQR 1.60 – 3.37)	2.41 (IQR 1.78 – 3.50)	0.77
Perforation			
- Yes	3 (17.6)	408 (16.5)	0.75
- No	14 (82.4)	2059 (83.5)	
Rate of complications Clavien Dindo grade III or higher	0 (0)	45 (1.8)	1.0
CCI®	1.73 ± 5.4	1.58 ± 6.6	0.93
Degree of inflammation (histologic examination)			
- No appendicitis	5 (29.4)	384 (15.6)	0.11
- Catarrhal	3 (17.6)	321 (13.0)	
- Suppurative	2 (11.8)	185 (7.5)	
- Suppurative with transmural ulcerations	4 (23.5)	1084 (43.9)	
- Gangrene / Perforation	3 (17.6)	408 (16.5)	
- Other	0	85 (3.4)	

Data are mean ± SD or *n* (%) or median (IQR)

*IQR* Interquartile range; *SD* Standard deviation; CCI® Comprehensive Complication Index

## Discussion

In this large retrospective series of woman who underwent emergency appendectomy for suspected appendicitis, the prevalence of appendiceal endometriosis was 0.7%, which is consistent with the findings of Chiou et al., who studied rare appendiceal lesions in patients who underwent emergent and nonemergent appendectomy and found a similar prevalence of appendiceal endometriosis (9 of 1134, 0.79%)[8].

This is 13 times higher than the prevalence estimated from autopsy series [10]. Interestingly, even in a highly selected population of woman with biopsy-proven endometriosis, appendiceal manifestation was less frequent than the prevalence in our study [20].

The higher incidence in our study compared to autopsy series and series of woman with previously diagnosed endometriosis raises the question whether the true incidence of appendiceal endometriosis is underestimated. A possible explanation for our findings is that a substantial number of patients with an appendiceal endometriosis undergo appendectomy at some point in their life for suspected appendicitis and do not require any further treatment for endometriosis in the future. Thus, these patients are underrepresented in studies investigating the anatomic distribution of endometriosis. This effect could be pronounced if endometriosis was indeed an underlying cause for appendicitis in some cases as has been postulated by some authors[12].

Appendiceal endometriosis seems to be more common in women with chronic right lower quadrant and pelvic pain. Gustofson et al. reported a prevalence of 3.7% for appendiceal endometriosis in patients with suspected endometriosis and chronic right lower quadrant pain and Agarwala & Liu found a prevalence of 4.4% in 317 woman who underwent appendectomy for chronic pelvic pain[1, 14]. Data on chronic or recurrent pain were not available for the patients in our study, but the duration of symptoms in patients with and without endometriosis did not differ between groups.

Although physical examination findings and laboratory results did not differ between groups, signs of appendicitis on ultrasound were less frequent in the endometriosis group although histopathologic examination revealed a similar rate of perforation. The data from this study do not allow conclusions to be drawn about whether endometriosis was a concomitant finding or an underlying cause of the inflammation, especially since the duration of symptoms, rates of perforation, complicated appendicitis, and complications did not differ between groups.

The complication rates in our study population (1.8%) correspond to those of a nationwide analysis of complications after appendectomy (2.1%), while the average length of hospital stay in our study was shorter than the national average [5, 19]. Complicated appendicitis was present in 60.3% of patients (*n* = 1499), which is comparable to the results of a German multicenter analysis [26]. The relatively high number of 15.5% negative appendectomies (appendix without signs of inflammation or endometriosis) could be due to the composition of our study population, which consisted only of women.

In recent years, randomized controlled trials have investigated the non-inferiority of antibiotic treatment compared to appendectomy for uncomplicated appendicitis [9, 23]. Although some studies showed non-inferiority of antibiotic treatment, two recent meta-analyses question these results [6, 15]. It should be considered that non-inflammatory pathologies of the appendix, such as appendiceal endometriosis, may remain unrecognized and continue to cause symptoms without surgical treatment.

This study has several limitations due to its retrospective nature. These include incomplete documentation, interpretation bias, and variability in the assessment of data and clinical management of patients. Ultrasound examinations and reports were carried out by a large number of radiologists with different levels of expertise. Furthermore, we were not able to collect data on the persistence of symptoms after appendectomy in endometriosis patients. Despite these limitations, this is one of the largest studies to investigate the incidence and clinical features of endometriosis in patients undergoing appendectomy for suspected appendicitis in an emergency setting.

## Conclusion

The prevalence of endometriosis in patients undergoing appendectomy for suspected appendicitis appears to be higher than suggested by data from autopsy series and even data from populations with biopsy-proven endometriosis, but lower than the prevalence found in patients with chronic or recurrent pelvic pain and suspected endometriosis. Patients with endometriosis of the appendix who present with acute pain are less likely to have a positive finding for appendicitis on ultrasound, but clinical and laboratory findings do not differ from those of patients without endometriosis.

## Data Availability

A fully anonymized data set can be made available upon justified scientific request and after ethical approval has been granted. Depending on the extent of the data use and the planned research, either appropriate credit or coauthorship must be granted to the authors of this study. Any requests should be addressed to the corresponding author.
